# Antifungal Activity of Essential Oil From *Zanthoxylum armatum* DC. on *Aspergillus flavus* and Aflatoxins in Stored Platycladi Semen

**DOI:** 10.3389/fmicb.2021.633714

**Published:** 2021-03-19

**Authors:** Ting Li, Mingyang Chen, Guangxi Ren, Guodong Hua, Jiu Mi, Dan Jiang, Chunsheng Liu

**Affiliations:** ^1^School of Chinese Materia Medica, Beijing University of Chinese Medicine, Beijing, China; ^2^Dongzhimen Hospital, Beijing University of Chinese Medicine, Beijing, China; ^3^Tibet University of Tibetan Medicine, Lhasa, China

**Keywords:** storage, aflatoxin, *Aspergillus flavus*, platycladi semen, essential oil, *Zanthoxylum armatum* DC.

## Abstract

The major objective of this study was to evaluate the inhibitory effect of essential oil (EO) from *Zanthoxylum armatum* DC. on *Aspergillus flavus*. The chemical composition of the EO was identified by gas chromatography–mass spectrometer. The minimum inhibitory concentration (MIC) of EO was investigated by liquid fermentation. The morphology, colony number, and aflatoxin content of *A. flavus* in platycladi semen were investigated by stereomicroscopy, scanning electron microscopy, plate counting, and high-performance liquid chromatography. The results indicated that the MIC of EO was 0.8 μL⋅mL^–1^, and the main components were β-phellandrene (7.53%), D-limonene (13.24%), linalool (41.73%), terpinen-4-ol (5.33%), and *trans*-nerolidol (6.30%). After the EO fumigated the platycladi semen, the growth of *A. flavus* slowed, and the mycelium shrank considerably. The number of colonies after EO treatment at room temperature and cold storage was significantly reduced, the inhibition effect was better under cold storage, and the aflatoxin B1 content did not exceed the standard within 100 days. Therefore, this study demonstrated the good potential of *A. flavus* growth inhibition during the storage of platycladi semen.

## Introduction

Aflatoxins are highly toxic secondary metabolites that are produced by several fungal species and that contaminate a wide variety of traditional Chinese medicines. Among them, aflatoxin B1 (AFB1), mainly produced by *Aspergillus flavus* and *Aspergillus parasiticus*, is the most significant form with respect to incidence and toxicity (Pellicer-Castell et al., 2020). AFB1 has carcinogenic, teratogenic, immunosuppressive, and other properties and was the first carcinogen listed by the International Cancer Agency (Marin et al., 2013; Songsamoe et al., 2017; Afsharmanesh et al., 2018; Chen et al., 2019). AFB1 is 68 times more toxic than arsenic and second only to botulinum. Therefore, it is of great interest to reduce aflatoxin contamination in Chinese medicinal materials.

Platycladi semen is a dried and mature seed of *Platycladus orientalis* (L.) Franco. It contains many cedrols, sitosterols, and terpenoids and a small amount of essential oil (EO) and saponins (Zhu et al., 2020). Modern pharmacological studies have shown that platycladi semen improves sleep and sedation, benefits intelligence, and protects nerves. It is mainly used clinically to treat insomnia, menorrhagia, constipation, night sweats, and other diseases (Zhu et al., 2020). However, during the storage of platycladi semen, aflatoxin is easily produced because of its rich oil (Li et al., 2019). The literature shows that aflatoxin contamination is still a threat to the quality and safety of platycladi semen (Chien et al., 2018). In view of the risk and harmful economic implications of aflatoxin contamination of platycladi semen, it is particularly important to solve the problem of aflatoxin contamination during the storage of platycladi semen.

Chemical fungicides were previously considered to be the most effective way to prevent the growth of *A. flavus* during the storage of medicinal materials. Recent studies have shown that chemical fungicides have a series of safety problems, such as teratogenicity, carcinogenesis, induced pathogen resistance, increased toxin production, and a long degradation cycle (Prakash et al., 2013; Gemeda et al., 2014). Therefore, to avoid a broader threat to the safety of medicinal materials, human beings, and ecosystems, the development of alternative fungal control methods with environmental safety and biodegradability is of great importance (Oliveira et al., 2020).

Essential oils are a good source of several bioactive compounds that possess antioxidative and antimicrobial properties (Cisarova et al., 2020). Some reports have described the antifungal activity of EOs (Belasli et al., 2020) and inhibition of aflatoxin synthesis (Mohammadi et al., 2020). However, there is no report on the prevention and treatment of *A. flavus* by EO during the storage of platycladi semen. In our previous studies, the EO of *Zanthoxylum armatum* DC. was selected among 16 kinds of plant EOs and could significantly inhibit the growth of *A. flavus* (Li T. et al., 2020). *Z. armatum* DC., a plant of Zanthoxylum in Rutaceae, is an important spice and Chinese medicinal material (Liu et al., 2020) that is expected to be a new type of botanical antibacterial agent. In this study, we further investigated the effects of the EO of *Z. armatum* DC. on the control of *A. flavus* growth and evaluated the potential use of the EO as a botanical antifungal agent during the storage of platycladi semen.

## Materials and Methods

### Materials

*Zanthoxylum armatum* DC. was purchased from Xiluyuan Market (Liangxiang, China). Platycladi semen was provided by Beijing Keyuan Xinhai Pharmaceutical Business Co., Ltd. All samples were identified by Prof. CL of Beijing University of Chinese Medicine and stored at −20°C. *A. flavus* was isolated from the surface of platycladi semen and preserved with glycerol solution at −80°C (Li T. et al., 2020). Aflatoxin standards were purchased from the Institute for Food and Drug Control (Beijing, China, lot no.: 61001-201703).

### Extraction and Characterization of the EO of *Z. armatum* DC.

The EO was obtained by hydrodistillation and stored in a brown sealed bottle at 4°C for use. The EO components were characterized by gas chromatography–mass spectrometer (GC-MS). The chromatographic column was HP-5 MS 5% phenyl methyl siloxane (0.25 mm × 30 mm × 0.25 mm). The initial temperature was 50°C, and the temperature was increased to 100°C at a rate of 3°C⋅min^–1^ and maintained for 3 min. Then, the temperature was increased to 240°C at a rate of 5°C⋅min^–1^ and maintained for 5 min. The carrier gas was He, the flow rate was 1.0 mL⋅min^–1^, the split ratio was 30:1, the gasification temperature was 250°C, and the solvent delay was 4 min. The electron energy was 70 eV, the ion source temperature was 270°C, and the mass range *m*/*z* was 50–600. The relative proportion of EO constituents was confirmed according to a database.

### Preparation of *A. flavus* Spore Suspension

*Aspergillus flavus* was periodically subcultured in PDA slants at 4°C and cultivated on PDA medium at 28°C for 7 days before use. Then, the *A. flavus* was washed with sterile water, and the mycelium was removed by filtration with sterile cotton. The suspension concentration of spores was adjusted to 2 × 10^–7^ cfu⋅mL^–1^ by counting on a hemocytometer.

### Minimum Inhibitory Concentration of the EO of *Z. armatum* DC.

Fifty milliliters of potato dextrose broth (PDB) medium at room temperature after sterilization was obtained, and an appropriate amount of the EO was prepared with Tween at concentrations of 0.02, 0.04, 0.08, 0.1, 0.2, 0.4, 0.6, 0.8, and 1 μL⋅mL^–1^. At the same time, 200 μL of *A. flavus* spore suspension with a concentration of 2 × 10^–7^ cfu⋅mL^–1^ was inoculated into the liquid culture medium and incubated at 120 rpm at 28 ± 0.5°C for 7 days. Each experiment was repeated three times. The mycelium were collected by vacuum filtration and dried to a constant weight, and the weight of the mycelium was measured to determine the minimum inhibitory concentration (MIC).

### Examination of the Morphology of *A. flavus*

The platycladi semen was disinfected first, and the steps were as follows: sterilization with 0.1% sodium hypochlorite solution for 1 min, rinsing with sterile water for 5 min and drying for 30 min. After that, the platycladi semen with 100 μL of *A. flavus* spore suspension on the surface was placed in a sterile culture dish, and the plate was covered with filter paper with an adhesive diameter of 2 cm. The EO was dripped on filter paper, and the same amount of sterile water was added as the control group. Each group was repeated three times, sealed, and cultured in the dark at 28°C for 7 days.

After fumigation with the EO for 7 days, the treated and control groups were first placed in 4% glutaraldehyde at 4°C overnight and rinsed three times with phosphate-buffered solution (0.1 M, pH 7.0) for 20 min each time. Gradient ethanol solution (20–100%, 10% intervals) was used to dehydrate the samples for 20 min each time (100% ethanol solution was rinsed for 30 min each time three times). The dried samples were observed under a stereomicroscope, and a small number of samples were sprayed with gold for scanning electron microscopy examination.

### Determination of *A. flavus* Colony Numbers

The surface-disinfected platycladi semen containing 100 μL of *A. flavus* spore suspension on the surface was placed in a sterile culture dish, and the EO was dripped onto sterile circular filter paper (2 cm) and adhered to the top of it. The control group was treated with the same volume of sterile distilled water on the dish, and dimethyl sulfoxide solution was used as the negative control group. The same batch of platycladi semen without any treatment was selected as the blank group. Each group was performed with three repetitions and placed at 20 and 4°C for culture, and the number of *A. flavus* was counted at 0, 2, 4, 6, and 8 days.

For the detection of colony number, 3 g of sample was transferred to a high-pressure conical flask containing 30 mL of sterile distilled water and homogenized for 15 min, and 200 μL of serial diluent was added to plate count agar (PCA) medium to determine the colony number. When the plate was covered with *A. flavus* and could not be counted, 60 colonies were recorded (Tang et al., 2018).

### Extraction and Determination of Platycladi Semen and Aflatoxin in Fumigation Storage of the EO

Five hundred grams of platycladi semen with known initial aflatoxin content was placed into a sealed bag (the initial group of platycladi semen was marked as O). Then, 0.5 mL of the EO was dropped into a sterile centrifuge tube with a small opening, which was placed in a plastic bag, sealed, stored at 4°C, and recorded as group A (i.e., the original medicinal materials plus EO). The same amount of sterile water was added to the same centrifuge tube as the control group, which was recorded as the B group (i.e., the original medicinal material group).

High-performance liquid chromatography (HPLC) was used to detect the aflatoxin content during a storage period. The method of the 2015 edition of the Chinese Pharmacopoeia was used as a reference for the extraction of aflatoxins (National Pharmacopoeia Commission, 2015). The liquid phase conditions were as follows: the mobile phase was methanol:acetonitrile:water (40:18:42), and detection was performed by post column derivatization. The flow rate of the derivatization pump was 0.3 mL⋅min^–1^, and the derivatization temperature was 70°C. A photochemical derivatizer (245 nm) was used. The excitation wavelength was 360 nm, and the emission wavelength was 450 nm.

### Statistics and Data Analysis

The dry weight of *A. flavus*, number of colonies, and mycotoxin concentrations in stored platycladi semen are expressed as the mean ± standard deviation. Statistical significance was evaluated using one-way analysis of variance (ANOVA) for multiple comparisons (SAS 9.4 Software). *P* < 0.05 was considered statistically significant.

## Results

### Analysis of the Minimum Inhibitory Concentration of EO From *Z. armatum* DC.

**FIGURE 1:**
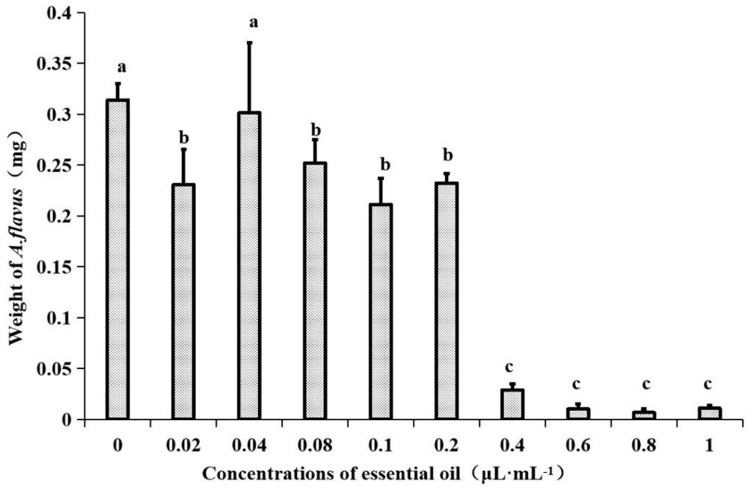
Effects of different concentrations of EO from *Z. armatum* DC. on mycelial weight in PDB media after 7 days of incubation at 28 ± 0.5°C. Values are the mean (*n* = 3) ± standard deviation. Different letters (a, b, c) indicate significant differences.

In a previous report, the plate fumigation method was used to study the antifungal activity of an EO. After 7 days of cultivation, no growth of *A. flavus* was found on the plate (Li T. et al., 2020). Therefore, MIC was determined by PDB liquid medium. The EO at different concentrations was dropped into PDB medium containing a certain concentration of *A. flavus* spore suspension. After 7 days of incubation, it was found that a large number of mycelial spherules appeared in PDB with concentrations of 0, 0.02, 0.04, 0.08, 0.1, and 0.2 μL⋅mL^–1^, whereas aseptic silk balls appeared in bottles with concentrations of 0.4, 0.6, 0.8, and 1 μL⋅mL^–1^, but the medium was slightly turbid. After ANOVA, as shown in Figure 1, there were significant differences among the EO concentrations of 0.4, 0.6, 0.8, and 1 μL⋅mL^–1^, and the dry weight of mycelium reached the minimum at the concentration of 0.8 μL⋅mL^–1^, so the MIC of the EO was determined to be 0.8 μL⋅mL^–1^.

### Chemical Composition Analysis of the EO From *Z. armatum* DC.

The EO of *Z. armatum* DC. was collected as a milky-white, oil-like liquid with a strong aroma, and the extraction rate was 4.07%. The results of GC-MS and database analysis are shown in Table 1. There were 66 components in the EO, accounting for 97.88% of the total EO. The main components of the EO were β-caryophyllene (7.53%), D-limonene (13.24%), linalool (41.73%), 4-terpenol (5.33%), and *trans*-nerolidol (6.30%). These major components could be the basis of alternative antifungal materials.

**TABLE 1:** Chemical composition of the EO from *Z. armatum* DC. identified by GC-MS.

	Retention	Percentage	
No.	time (min)	(%)	Compounds
1	7.21	0.21	3-Thujene
2	7.47	0.86	α-Pinene
3	8.02	0.03	Camphene
4	9.46	7.53	β-Phellandrene
5	10.11	1.86	β-Pinene
6	10.57	0.32	α-phellandrene
7	11.18	0.57	1,3-Cyclohexadiene,1-methyl-4- (1-methylethyl)
8	12.46	13.24	D-Limonene
9	12.58	0.09	*trans*-β-Ocimene
10	13.04	0.45	β-Ocimene
11	13.65	1.94	γ-Terpinene
12	14.27	0.04	*trans*-4-Thujanol
13	15.24	0.73	Terpinolene
14	19.41	41.73	Linalool
15	19.57	0.27	2-Cyclohexen-1-ol,1-methyl-4- (1-methylethyl)-, (1*R*,4*S*)-rel-
16	21.17	0.05	Borneol
17	22.04	5.33	Terpinen-4-ol
18	22.69	2.22	α-Terpineol
19	22.87	0.50	6,6-Dimethyl-bicyclo(3.1.1) hept-2-ene-2-methanol
20	23.32	0.19	2-Cyclohexen-1-ol,3-methyl-6- (1-methylethyl)-, (1R,6S)-rel-
21	24.55	0.28	Nerol
22	24.99	0.13	Cuminaldehyde
23	25.22	0.06	D-Carvone
24	25.83	0.19	Piperitone
25	26.19	0.75	Geraniol
26	26.97	0.08	Phellandral
27	27.76	0.45	Anethole
28	28.11	0.06	Cuminol
29	28.30	0.04	2-Undecanone
30	28.99	0.03	*p*-Thymol
31	29.46	0.03	2-Methoxy-4-vinylphenol
32	30.08	0.08	(1s)-6,6-Dimethylbicyclo(3.1.1) hept-2-ene-2-methanol acetate
33	30.74	0.06	Dodecamethylcyclohexasiloxane
34	30.93	0.02	3,7-Dimethyl-6-octenoic acid
35	31.54	0.10	Terpinyl acetate
36	32.62	0.04	Nerol acetate
37	32.91	0.05	α-Cubebene
38	33.03	0.24	β-Elemen
39	35.60	1.08	Caryophyllene
40	37.30	0.76	Humulene
41	37.69	0.05	β-Bisabolene
42	38.62	1.33	1,6-Cyclodecadiene,1-methyl-5-methylene-8- (1-methylethyl)-, (1*E*,6*E*,8*S*)-
43	38.76	0.37	Naphthalene
44	39.20	0.66	3,7,11,11-Tetramethylbicyclo(8.1.0) 2,6-undecadiene
45	39.36	0.06	α-Muurolene
46	39.48	0.13	1-Methyl-2,4-di(prop-1-en-2-yl)-1-vinylcyclohexane
47	39.85	0.19	γ-Muurolene
48	40.24	0.27	D-Cadinene
49	41.17	0.33	Cyclohexanemethanol,4-ethenyl-α,α,4-trimethyl-3- (1-methylethenyl)-, (1*R*,3*S*,4*S*)-
50	42.11	6.30	*trans*-Nerolidol
51	42.23	0.39	Caryophyllene oxide
52	42.83	0.06	Hexadecane
53	43.40	0.13	γ-Eudesmol
54	43.74	0.25	Uncineol
55	44.05	0.64	*g*-Cadinene
56	44.29	0.29	β-Eudesmol
57	44.42	0.57	α-Cardinol
58	44.86	0.16	2-Hydroxy-4,6-dimethoxyacetophenone
59	46.13	0.12	*trans*-Farnesol
60	48.88	0.08	Farnesol acetate
61	51.20	0.51	Palmitoleic acid
62	51.87	1.47	Palmitic acid
63	53.02	0.08	Ethyl palmitate
64	54.02	0.02	Methyl linolenate
65	55.01	0.67	11-*cis*-Vaccenic acid
66	55.26	0.07	Ethyl linolenate
		97.88	

### Morphological Effects of the EO of *Z. armatum* DC. on *A. flavus*

**FIGURE 2:**
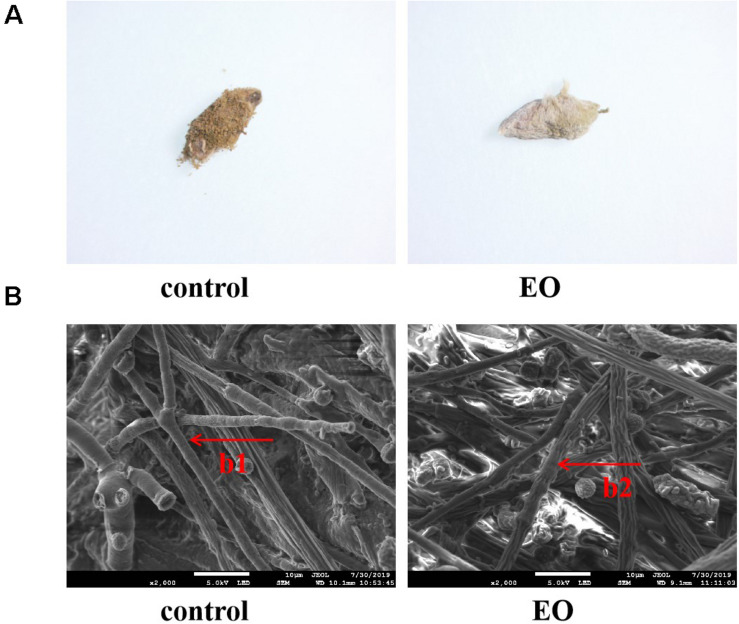
Morphology of *A. flavus* on the surface of platycladi semen treated with the EO after 7 days’ incubation under microscope **(A)** and scanning electron microscope **(B)** (b1: rod-shaped and smooth mycelium; b2: irregular shrinkage mycelium).

A stereomicroscope was used to observe the morphology of platycladi semen with *A. flavus* attached after EO and sterile water treatment (Figure 2A). The mycelium of *A. flavus* fumigated by EO was light yellow after 7 days, the mycelial shrinkage was visible as destruction, and the growth was slow. However, *A. flavus* in the control group was yellow-green, with a large number of granules on the surface, and grew vigorously. Scanning electron microscopy showed that the morphology of *A. flavus* after fumigation with the EO group and control group was very different (Figure 2B). In the control group, a large number of plump mycelia covered the surface of the platycladi semen, and the mycelium was rod-shaped and smooth. However, the mycelium on the surface of platycladi semen with EO was less abundant, and irregular shrinkage appeared. These findings were similar to those observed in earlier reports (Li et al., 2016). It is speculated that the EO of *Z. armatum* DC. may damage the growth of mycelium and inhibit *A. flavus*.

### Effect of the EO of *Z. armatum* DC. on the Colony Number of *A. flavus*

**FIGURE 3:**
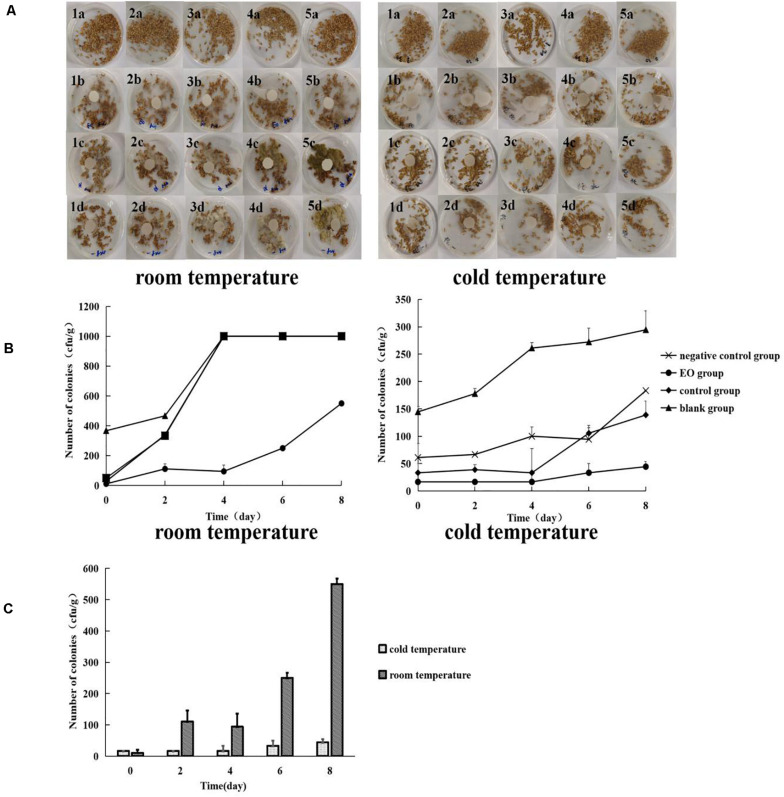
Growth and colony number of *A. flavus* on the surface of platycladi semen treated with EO at room temperature (20°C) and cold storage (4°C) [**(A)** growth of *A. flavus* on the surface of platycladi semen; a: blank group; b: EO group; c: control group; d: negative control group; 1, 2, 3, 4, and 5 indicated the growth of *A. flavus* on the surface of platycladi semen on day 0, 2, 4, 6, and 8; **(B)** the number of colonies of *A. flavus* on the surface of *Platycladus orientalis* at 0, 2, 4, 6, and 8 days; **(C)** colony number of *A. flavus* in the EO group at room temperature (20°C) and cold storage (4°C) at 0, 2, 4, 6, and 8 days.

At room temperature, no visible *A. flavus* appeared on the surface of platycladi semen at the beginning of each group. As the incubation time increased, on the seventh day, the negative control group and control group clearly showed the growth of *A. flavus* on the surface of platycladi semen. A small amount of *A. flavus* was visible on the surface of platycladi semen in the EO group, whereas no *A. flavus* adhesion was observed on the surface of each group of samples under cold storage condition (Figure 3A). After incubation, the number of *A. flavus* colonies on the surface of platycladi semen was counted (Figure 3B). At room temperature, it can be seen that except for the EO group, which showed an upward trend in the number of *A. flavus* colonies after 4 days, the number of *A. flavus* colonies in the other groups demonstrated an upward trend at 2 days and reached the maximum value at 4–6 days; that is, *A. flavus* could be seen growing on the whole plate. Similarly, under cold storage, the blank group, negative control group, and control group showed an upward trend. Compared with these groups, the number of *A. flavus* was almost the lowest in the EO group, and only an average colony number of 44.44 cfu⋅g^–1^ was detected. This indicates that the EO under cold storage can effectively inhibit *A. flavus*, suggesting that it can be used as a new type of antifungal agent during the storage of platycladi semen.

For the fumigation of the EO under the two different conditions, room temperature 20°C and cold temperature 4°C, the changes in *A. flavus* colony number on the surface of platycladi semen are shown in Figure 3C. It can be seen that the growth of *A. flavus* on the surface of platycladi semen under cold storage conditions was inhibited relative to that under room temperature conditions, so fumigation with EO under cold storage conditions is more effective.

### Effect of the EO of *Z. armatum* DC. on Aflatoxin Content in Platycladi Semen

The results of HPLC and database analysis are shown in Table 2. When platycladi semen was obtained from the market, the results showed that the content of AFB1 was 0.45 μg⋅kg^–1^, and the total content of aflatoxin (sum of AFB1, AFB2, AFG1, AFG2) was 0.57 μg⋅kg^–1^. Aflatoxins were monitored after storage for a period of time. On the 10th day, the aflatoxin content in the two groups did not change much. The content of AFB1 in the EO group reached 0.44 μg⋅kg^–1^, and the total content was 0.57 μg⋅kg^–1^, whereas the content of AFB1 in the control group reached 1.45 μg⋅kg^–1^, and the total content reached 2.64 μg⋅kg^–1^. On the 100th day of storage, the aflatoxin content in the two groups increased significantly. The content of AFB1 in the EO group reached 2.28 μg⋅kg^–1^, and the total content was 2.77 μg⋅kg^–1^, whereas the content of AFB1 in the control group reached 6.84 μg⋅kg^–1^ [the content exceeded the limit of pharmacopeia (≤5 μg⋅kg^–1^)] (National Pharmacopoeia Commission, 2015).

**TABLE 2:** Detection of aflatoxin production by *A. flavus* in platycladi semen with the EO by HPLC ($\bar{x}$ ± s, *n* = 3).

Storage time (days)	Group	Content (μg ⋅ kg^–1^)				
		AFB1	AFB2	AFG1	AFG2	Total
0	O	0.45 ± 0.02 d	0.12 ± 0.01 d	N	N	0.57 ± 0.02 e
10	A	0.44 ± 0.02 d	0.13 ± 0.01 d	N	N	0.57 ± 0.03 d
	B	1.45 ± 0.02 c	1.07 ± 0.13 b	N	0.12 ± 0.03 a	2.64 ± 0.14 c
100	A	2.28 ± 0.00 b	0.49 ± 0.01 c	N	N	2.77 ± 0.02 b
	B	6.84 ± 0.15 a	2.00 ± 0.17a	N	0.02 ± 0.02 b	8.86 ± 0.05 a

*Different letters in the same column indicate significant differences, *P* < 0.05; “N” means not detected; O, initial group of platycladi semen; A, original medicinal materials plus EO; B, original medicinal material group.*

## Discussion

Contamination with fungi and subsequent mycotoxins is regarded as one of the world’s most severe problems (Williams et al., 2004) and has attracted notable attention. To eliminate this contamination, many methods are used to prevent fungal growth or inhibit the production of mycotoxins. At present, the use of plant EO to inhibit the growth of *A. flavus* and then inhibit aflatoxin contamination is a very effective method (Mittal et al., 2019). EO has unique advantages in the prevention and control of *A. flavus* (Prakash et al., 2015). The first is safety. Most EOs come from plants with the same medicinal and edible origins, such as mint, cinnamon, and dried ginger. The second is volatility, which makes its application extremely simple. The EO only needs to be placed in a closed space. It is not only easy to contact *A. flavus* but also to maintain a high concentration of EO for a long time. In addition, a small dose of EO can be used for fumigation to achieve antifungal activity.

In this study, we found that the EO of *Z. armatum* DC. has high antifungal activity, which can destroy the growth of fungal hyphae, reduce the number of *A. flavus* colonies and the toxin content, and extend the storage period of platycladi semen. These findings suggest that EOs represent good candidates for controlling toxigenic fungi and subsequent mycotoxins.

Gas chromatography–mass spectrometer analysis revealed that the main components were β-caryophyllene (7.53%), D-limonene (13.24%), linalool (41.73%), 4-terpenol (5.33%), and *trans*-nerolidol (6.30%). The main components of the EO were slightly different from those reported in the literature (Nooreen et al., 2019), such as linalool, isohexanal, and methyl-10-octadecanoate, which may be caused by the genetic differences of different plant populations, environmental and soil limitations, harvesting time, drying methods, and inconsistent extraction and analytical methods (Prakash et al., 2015; Nea et al., 2020). The biological activity of EO depends on the combined action of its main and minor components because even a small change in the content of EO may change its antifungal activity (Li Y. et al., 2020). Therefore, it is necessary to characterize the chemical properties of EO before determining its antifungal activity.

The 2015 edition of the Chinese Pharmacopoeia stipulated that the storage conditions of platycladi semen must be cool and dry (National Pharmacopoeia Commission, 2015). However, through investigation in the early stage of this study, it was found that storage of platycladi semen more commonly occurred at room temperature. The results showed that the colony number of *A. flavus* was 366.67 cfu⋅g^–1^ at room temperature and 144.44 cfu⋅g^–1^ under cold storage, indicating that *A. flavus* was not active or grew slowly on platycladi semen under low temperature. Therefore, the refrigeration method is preferred for the storage of platycladi semen. Furthermore, the results showed that the number of colonies with EO fumigation at room temperature or cold storage was lower than that in other treatment groups, suggesting EO is a potential natural antifungal agent.

The aflatoxin content of platycladi semen treated with EO during storage was tested, and the results showed that the EO of *Z. armatum* DC. could prolong the storage period of platycladi semen and ensure that aflatoxin did not exceed the standard within 100 days. Therefore, considering economic security and other factors and aflatoxin contamination in platycladi semen, the following prevention measures are proposed: when the aflatoxins in the initial storage of platycladi semen did not exceed the limit, the EO of *Z. armatum* DC. could effectively inhibit *A. flavus*, thus slowing down the production time of aflatoxin and extending the storage time. Therefore, for medicinal materials that are susceptible to aflatoxin, prevention and control of the growth of *A. flavus* and its toxin contamination are very important.

## Conclusion

To evaluate the application prospects of the EO from *Z. armatum* DC. on *A. flavus* in stored platycladi semen, the inhibition efficiency of the EO on fungal morphology, colony number, and mycotoxin production was evaluated using stereomicroscopy, scanning electron microscopy, plate counting, and HPLC. When the EO was treated with platycladi semen, we found that the EO could destroy the hyphae of *A. flavus* and greatly reduce the number of colonies in the stored platycladi semen. Moreover, the EO had the best antifungal effect under cold storage conditions. In addition, EO can extend the storage time of platycladi semen, and the aflatoxin B1 content did not exceed the standard within 100 days. These findings provide substantial solid evidence for the successful application of EO from *Z. armatum* DC. to control toxigenic fungi and subsequent mycotoxin contamination during storage.

## Data Availability Statement

The original contributions presented in the study are included in the article/supplementary material, further inquiries can be directed to the corresponding author/s.

## Author Contributions

TL and DJ participated in the design of the study and data analysis, and prepared the manuscript. TL and MC conducted the experiments. GR, GH, and JM participated in the design of the study. CL was responsible for the overall supervision of the work. All authors read and approved the final manuscript.

## Conflict of Interest

The authors declare that the research was conducted in the absence of any commercial or financial relationships that could be construed as a potential conflict of interest.
